# Two Cases of Post-Partum Hemorrhage Treated by the Injection of Perchloride of Iron

**Published:** 1875-12

**Authors:** W. P. Swain


### TWO CASES OF POST-PARTUM HEMORRHAGE TREATED BY THE INJECTION OF PERCHLORIDE OF IRON.

BY W. P. SWAIN, F. R. C. S.

Without wishing to fan into a flame the dying embers of controversy on the subject of the injection of perchloride of iron in post-partum hemorrhage, I desire to put on record the two following cases which have occurred in my practice during the last six months:

Case 1 was that of an exceeding fine, handsome lady, a primipara. Her pregnancy had been uninterruptedly good. Labor commenced on the evening of January 21st. The presentation was normal, the pains good, and a fine male child was born about half-past seven on the morning of the 22d. I gave a small quantity of chloroform at the last. Immediately after the delivery of the child I found a second head presenting, and, as the pains were not very expulsive, I put on the forceps and delivered a second male child in a few minutes. I then removed the placenta, which was single and of enormous size. Considerable hemorrhage occurred at the moment the placenta was expelled, but the womb contracted firmly, and a binder was put on. Within a few moments, however, I found the uterus largely expanded, and on pressure a huge clot was expelled. I immediately introduced my left hand into the uterus, making pressure externally with the right, but was unable to produce any uterine contraction. During the whole time there was a constant and excessive flow of blood from the vagina, and the patient became collapsed. I administered brandy in large quantities and ergot, and the moment ice could be obtained placed a large lump in the cavity of the womb, and a bag of ice on the abdomen. All was, however, of no avail, and my hopes of saving the patient were at zero. In the meantime I had obtained further professional asssitance, and, with the concurrence of my father and Mr. Whipple, I injected perchloride of iron into the uterus, in the manner advised by Dr. Barnes. From the moment of the injection all flow of blood ceased, although the uterus remained for some time flaccid. The lady made an excellent recovery, and, with the exception of a little lymphatic tenderness in the right thigh, complained of nothing during her convalescence. I should mention that on the second day, and on every succeeding day for some time, the vagina was thoroughly well syringed out with Condy's fluid and water.

Case II.—I was asked by Surgeon-Major Ferguson to see at the Woman's Hospital, on July 9th, a soldier's wife, in her third pregnancy. She had a contracted pelvis in its antero-posterior diameter. In her first confinement she remained a hundred and forty-eight hours in labor; in her second, eighty-seven hours. No instrumental assistance was afforded on either occasion, and the children were still-born. This time she had been in labor sixty hours, and I found the head well down on the pelvis but tightly fixed there. I suggested the application of the forceps, and in a very short time, after the use of considerable traction, the child was born alive, but lived only for half an hour. The placenta was removed at once without difficulty, and the uterus contracted for a short time, but in a few moments tremendous hemorrhage set in, tho blood being projected from the vagina on the floor some distance from the bed. I immediately passed my left hand into the uterus, cleared out its contents, and endeavored to secure contraction, whilst cold water was poured on the abdomen from a height, and brandy freely administered. This, however, did not correct the bleeding, although it somewhat lessened in quantity. Feeling that another gush of hemorrhage might be fatal, I injected the perchloride, all the materials being fortunately at hand. The uterus at once contracted, all hemorrhage ceased, and I hear from Dr. Ferguson that the woman has made an admirable recovery.

I think I may fairly claim two lives for the perchloride treatment. In the first case, all the usual resources, excepting galvanism, were tried in vain; the lady lay dying before our eyes, and there can be no doubt that any further hemorrhage must have turned the balance against her. In the second case, so rapid and large was the loss of blood, that nothing short of immediate arrest could have saved the patient; and this the perchloride certainly effected.—*Obstetrical Journal.*

